# RNA sequencing of mRNA 5’-ends reveals regulators of bovine embryonic genome activation

**DOI:** 10.1186/s12864-025-12110-x

**Published:** 2025-10-14

**Authors:** Barış Yaşar, Nina Boskovic, Tõnis Org, Juha Kere, Ants Kurg, Shintaro Katayama

**Affiliations:** 1 Department of Biotechnology, Institute of Molecular and Cell Biology, University of Tartu, Tartu, 51010 Estonia; 2 Department of Medicine Huddinge, Karolinska Institutet, Huddinge, 14183 Sweden; 3 Department of Obstetrics and Gynecology, University of Helsinki, Helsinki, 00290 Finland; 4 Centre for Genomics, Evolution and Medicine, Institute of Genomics, University of Tartu, 51010 Tartu, Estonia; 5 Folkhälsan Research Center, Helsinki, 00290 Finland; 6 Stem Cells and Metabolism Research Program, University of Helsinki, Helsinki, 00290 Finland

**Keywords:** Preimplantation embryo development, RNA sequencing, Bovine, Reproduction, Homeodomain, PRD-like (PRDL) homeobox gene

## Abstract

**Background:**

The maternal-to-zygotic transition (MZT) in embryo development involves the degradation of maternal transcripts and the initiation of zygotic transcription, known as embryonic genome activation (EGA). EGA is marked by the upregulation of several PRDL homeobox family genes in humans. Many orthologues of these genes have recently been identified in bovine based on a comparative analysis with human EGA. Although studies on bovine EGA exist, a comprehensive analysis integrating 5’ end mRNA sequencing with PRDL homeobox gene annotations based on cDNA evidence from bovine early embryo development is still lacking.

**Results:**

This study employs STRT-N RNA sequencing (RNA-seq) to profile the 5’ ends of transcripts in single bovine oocytes and preimplantation embryos, identifying transcription factors (TFs) involved in EGA. We observed significant transcript degradation at the 2- and 16-cell stages, with the latter marking the onset of EGA. Our analysis characterized bovine EGA through the transient upregulation of PRDL homeobox family genes, similar to human EGA, and by identifying several marker genes. Furthermore, promoter analysis revealed potential EGA-inducing TFs, including PRDL homeodomain (DUXA, PHOX2A, UNCX, ALX1, LEUTX, and TPRXs) and other TFs (GABPA, EBF1, RELA, and KLFs).

**Conclusions:**

Our findings provide a comprehensive view of the dynamic transcriptome regulation during bovine preimplantation development and highlight key regulatory elements and TFs that may drive EGA.

**Supplementary Information:**

The online version contains supplementary material available at 10.1186/s12864-025-12110-x.

## Background

The maternal-to-zygotic transition (MZT) involves the transfer of developmental control from the mother to the embryo, characterized by the degradation of maternal transcripts and the initiation of zygotic transcription [1]. Early zygotic transcription, which includes a burst of RNA synthesis from the previously silenced zygotic genome, is termed embryonic genome activation (EGA) [2]. The timing of EGA is species-specific [3], occurring between the mid 1-cell and late 2-cell stages in mice, the 4-cell and 8-cell stages in humans, and the 8-cell and 16-cell stages in bovine in vitro fertilization (IVF) embryos [4, 5]. The degradation of maternal transcripts is essential for EGA [6], and recent studies suggest it is governed by various mechanisms [7].

Paired-like (PRDL) proteins belong to the homeodomain protein class, conserved across bilaterians, and act as transcription factors (TFs) involved in gene expression regulation [8]. Our previous transcriptomic studies have shown that many PRDL homeobox TF genes are exclusively expressed in human early embryos [9, 10]. In bovine, transcriptomic studies across several early developmental stages have been conducted [3, 5, 6, 11–15]. These studies, however, have not detected several PRDL homeobox family genes recently identified through comparative analysis with human EGA [16]. Based on their temporal activation, PRDL homeobox family genes can be categorized as maternal and zygotic genes [9]. While maternal PRDL homeobox genes are conserved across many species, including non-vertebrates [9], zygotic genes exhibit variability even within placental mammals [17, 18]. These fast-evolving genes are thought to function with species-specific developmental requirements, such as EGA timing or development speed. Therefore, further investigation of PRDL homeobox genes requires a specific focus on the transcriptome during bovine preimplantation development.

RNA sequencing (RNA-seq) methods vary in their ability to produce greater read depth in specific regions of the gene: full-length, 3’ end, or 5’ end [19]. The latter shows coverage peak at the 5’ ends of genes [20], enabling exploration of promoter activity and analysis of promoter regulatory elements. Regulatory elements are specific DNA sequences recognized by TFs, modulating transcriptional programs [21]. RNA-seq methods focusing on the 5’ end allow reliable investigation of transcription start site (TSS) and gene promoters, aiding in the study of proximal regulatory elements. Quantification strategies in such RNA-seq methods prioritize the 5’ ends of genes [22], ensuring that only transcripts with intact 5’ ends, and thus functional transcripts, are counted. This promoter-based approach allows the quantitation of novel transcripts and prevents noise from degrading transcripts or those lacking coding potential.

The present study aims to identify TFs, including the rapidly evolving PRDLs, contributing to bovine EGA using STRT-N RNA-seq [23] in single bovine oocytes and preimplantation embryos. This method focuses on the 5’-end profiling, and relies on poly(T) priming for complementary DNA (cDNA) synthesis for quantifying poly(A)-tailed RNAs, template switching for capturing the 5’ ends of the RNAs, and unique molecular identifier (UMI) and external spike-in RNA addition for accurate expression profiling without prior knowledge of endogenous control genes [23]. Further, as in our previous study of human preimplantation development [9], we employed transcript far 5’-end (TFE), which is a promoter-based quantification unit. With our TFE-based analysis, we explored differential expression between consecutive stages and confirmed 8-to-16-cell transition to be the time of EGA for bovine IVF embryos [5, 12]. Furthermore, we showed with our TFE data that this transition, similar to 4-to-8-cell transition in human [9], is the time when several recently characterized PRDL homeobox TF genes [16], namely *ARGFX*, *DUXA*, *LEUTX*, and *TPRX*s, are transiently upregulated. We also identified candidate DNA sequence motifs enriched in promoters around the 16-cell stage-specific TFEs. These motifs can be targeted by PRDL class (DUXA, PHOX2A, ALX1, UNCX, LEUTX and TPRXs) and other class TFs (GABPA, EBF1, RELA, and KLFs), whose mRNAs were detected in early bovine embryos, implying their potential roles in promoting EGA. By taking a promoter-targeted approach, our study offers a revised and updated perspective on the dynamic transcriptome regulation of bovine preimplantation development.

## Results

### Promoter-based expression profile showed EGA-centered clustering of embryonic stages

**Fig. 1:**
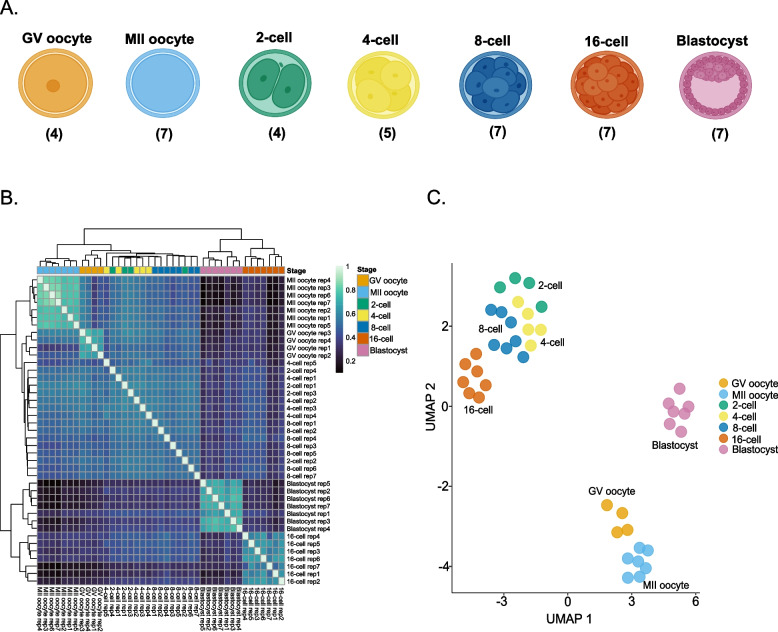
Summary of the RNA-seq sample composition and clustering. **A** Illustration of the bovine developmental stages used for STRT-N RNA-seq. **A** was created with BioRender. **B** Heatmap showing the correlation of RNA-seq samples based on the most variable 20,000 TFEs. **C** UMAP plot of all samples

To investigate bovine MZT at transcriptional level, we collected single oocytes and IVF embryos from the GV and MII oocytes, and the 2-, 4-, 8-, 16-cell and blastocyst stages (Fig. 1A) and analyzed them using STRT-N RNA-seq method [23]. Sequenced RNA-seq libraries consisted of 13 oocytes, 33 preimplantation stage embryos, and two no-RNA (negative) control samples. Three samples (11, 13, and 26) that showed low mapping rates, two outliers (samples 23 and 37) and negative controls (1 and 41) were eliminated from the downstream analyses (Supplementary Fig. 1; Supplementary Table 1). We retained all blastocyst samples as the mapped reads for sample 42 were in the acceptable range even though the mapped rate was slightly lower than the threshold (Supplementary Fig. 1). The RNA-seq samples had a minimum of 1,161,533 reads and a maximum of 8,890,559, with the median read number being 2,806,839 (Supplementary Table 1). The median of the mapping rates for all libraries was 60.1% (Supplementary Table 1). We detected 122,688 TFEs, with 34,331 of them being unannotated, whereas 88,357 TFEs were associated with 18,501 known genes.

For comparing the expression profiles between stages and replicates, we calculated pairwise correlation of samples using top 20,000 most variable TFEs, which revealed two main clusters distinguishing pre- and post-EGA developmental stages (Fig. 1B). Out of the top 20,000 TFEs, 9,778 were unannotated and 10,222 TFEs matching annotated features were associated with 6,695 genes. All MII oocyte, 16-cell and blastocyst samples grouped separately, the MII stage showing near-zero correlation with other two later stages. On the heatmap, GV oocyte replicates grouped together showing higher correlation with 2-cell, 4-cell and 8-cell stage embryos, which did not separate as distinctly, suggesting similar transcriptional profiles of IVF embryos before EGA (Fig. 1B). Similar results were seen in the UMAP plot, in which each developmental stage formed a distinct cluster while cleavage stages grouped into one broader group acting as a transitioning state (Fig. 1C).

### Relative poly(A)-tailed RNA content change and genomic annotations of TFEs in the profile reflected MZT

**Fig. 2:**
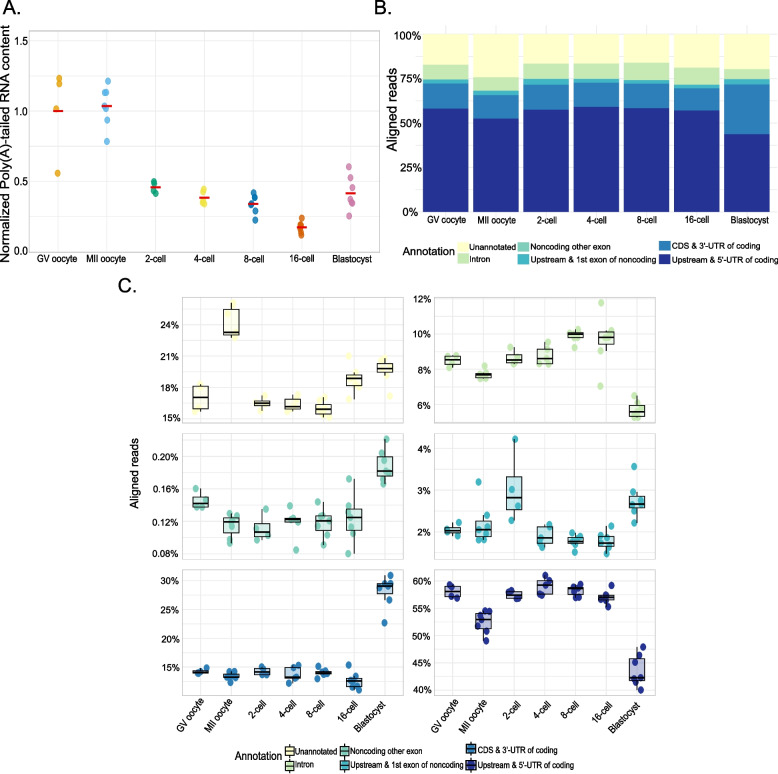
RNA content and genomic location annotation. **A** Normalized total number of mapped reads per sample averaged by stage with respect to GV oocyte that was set to one. **B** Proportions of mapped reads averaged by stage based on their genomic position relative to the annotated genes. **C** Detailed representation of **B** showing the percentage of each annotation across stages

To characterize bovine MZT in poly(A)-tailed RNAs globally, we estimated total poly(A)-tailed RNA content relative to the spike-in RNAs in each stage. Changes of total poly(A)-tailed RNA levels between consecutive developmental stages demonstrated steady levels during oocyte maturation, after which a continuous decrease until the 16-cell stage was observed (Fig. 2A). The decrease was reversed at the blastocyst stage, where the poly(A)-tailed RNA content increased to more than double of that found in the 16-cell stage.

To further characterize bovine MZT over the developmental stages, we calculated the proportions of averaged aligned reads based on their genomic features, identifying TFEs that correspond to portions of coding (upstream and 5’-UTR or coding sequence [CDS] and 3’-UTR), noncoding (upstream and 1 st exon or exons other than 1 st) genes as well as introns and unannotated regions of the genome, with regard to the reference bovine gene model with 36,075 gene definitions (Ensembl [24] Release 112; Fig. 2B & C; Supplementary Table 2). The share of known coding genes was close to 75% in most stages, being slightly lower in the 16-cell stage and even lower in the MII oocyte. The percentages of TFEs aligning to the upstream and 5’-UTR of coding genes decreased and those aligning to the CDS and 3’-UTR of coding genes increased at the blastocyst stage, possibly as a result of degrading mRNA molecules.

### TFE-based differential expression analysis showed bovine EGA timing and major waves of RNA degradation

**Fig. 3:**
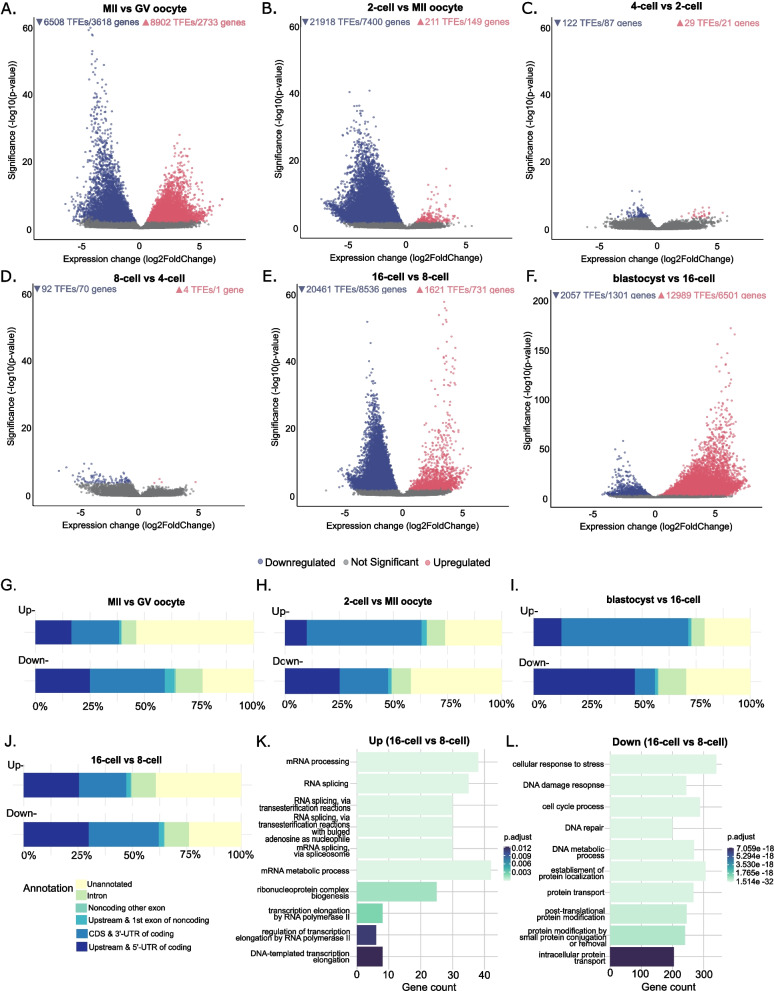
Differentially expressed TFEs and corresponding genes, their genomic annotation and possible biological functions. **A**-**F** Up- (reddish-pink) and downregulated (deep blue) TFEs/genes are shown in a volcano plot. Each developmental stage is compared to the stage preceding it. **G-J** The genomic features of differentially expressed TFEs in (**A**), (**B**), (**E**), and (**F**) are shown as 100% stacked bar charts in (**G**), (**H**), (**I**), and (**J**), respectively. **K**, **L** The top 10 significant biological process (BP) gene ontology terms are shown for differentially expressed genes in (**E**)

We compared the expression of individual TFEs at each developmental stage to the preceding one to identify genes with notable changes that may indicate their roles in early embryo development. Progression from the GV to MII oocyte was accompanied with the upregulation of 8,902 and the downregulation of 6,508 TFEs (Fig. 3A). Transition from the MII oocyte to the 2-cell stage embryo was characterized by the mild upregulation of 211 and massive downregulation of 21,918 TFEs (Fig. 3B). Development into the 4- and 8-cell stages resulted in the downregulation of 122 and 92 TFEs, respectively, whereas the upregulation remained minimal with 29 TFEs in the 4-cell and four TFEs in the 8-cell stages (Fig. 3C, D). Eight-to-16-cell transition uncovered upregulation of 1,621 TFEs, which marked the highest degree of activation among the consecutive cleavage stages, marking this transition as the time of bovine EGA (Fig. 3E; Supplementary Table 3). The second most pronounced downregulation, comprising 20,461 TFEs, was observed during this transition (Fig. 3E; Supplementary Table 4). Development into the blastocyst stage showed 12,989 upregulated TFEs whereas 2,057 of the TFEs were downregulated (Fig. 3F). Altogether, these results present a general summary of the events occurring in bovine early development including EGA and MZT.

More than half of the upregulated TFEs between two stages of oocyte were unannotated pointing to unexplored factors in bovine oocyte maturation (Fig. 3G). During this process downregulated coding transcripts made up almost 60% of all downregulated transcripts. The most significant GO term for upregulated TFEs was cell cycle process, which is self-evident for the progression of prophase I-arrested GV oocytes to resume meiosis [25] (Supplementary Fig. 2 A). The top GO terms for downregulated transcripts were translation and peptide biosynthetic process.

Following fertilization on progression to the 2-cell stage embryo, we observed that 63% of upregulated TFEs were of coding genes, out of which 53% were annotated as 3’-UTR and CDS, and 10% as 5’-UTR and upstream (Fig. 3H). The majority of the downregulated TFEs were found to be unannotated, a high percentage of which may be derived from the high representation of unannotated transcripts in MII oocytes as a result of maternal mRNA degradation (Figs. 2B and 3H). GO terms for upregulated TFEs with high significance were related to nucleosome assembly and chromatin remodeling, processes that might contribute to the loosely organized chromatin of the 2-cell stage [26] (Supplementary Fig. 2B).

Bovine EGA during 8-to-16-cell transition was marked by upregulation of several transcripts, close to half of which were coding (Fig. 3J). Of these coding transcripts 22% had their TFEs in the coding sequence or 3’-UTR of coding genes, whereas 25% of them had their TFEs in the 5’-UTR or even more upstream region of the coding genes. Interestingly, 39% of the upregulated TFEs were unannotated, suggesting the possibility of containing critical yet unexplored players in bovine EGA. For upregulated TFEs, we observed GO terms related to transcription-coupled processes in line with the major activation of the embryonic genome (Fig. 3K). Top GO terms for downregulated TFEs were cellular response to stress and DNA damage response, suggesting a shift to priority for fundamental cellular processes (Fig. 3L).

During the 16-cell-to-blastocyst transition, we detected the highest number of upregulated transcripts, 59% of which are comprised of TFEs in the CDS or 3’-UTR of coding genes (Fig. 3I). Conversely, downregulated TFEs in the 5’-UTR or upstream of available coding gene models, were more represented, making up 47% of all downregulated transcripts (Fig. 3I). GO term analysis of upregulated TFEs for this stage included cellular response to stress that is common for embryos exposed to longer culturing [27] and low oxygen [28] (Supplementary Fig. 2C).

### Transient upregulation of several PRDL homeobox genes at the 16-cell stage and unexplored transcription start sites

As a further investigation of dynamic expression changes in PRDL homeobox genes during the bovine development, we overlaid these loci with the cDNAs isolated in our previous study [16] and the TFEs identified in the present study.

**Fig. 4:**
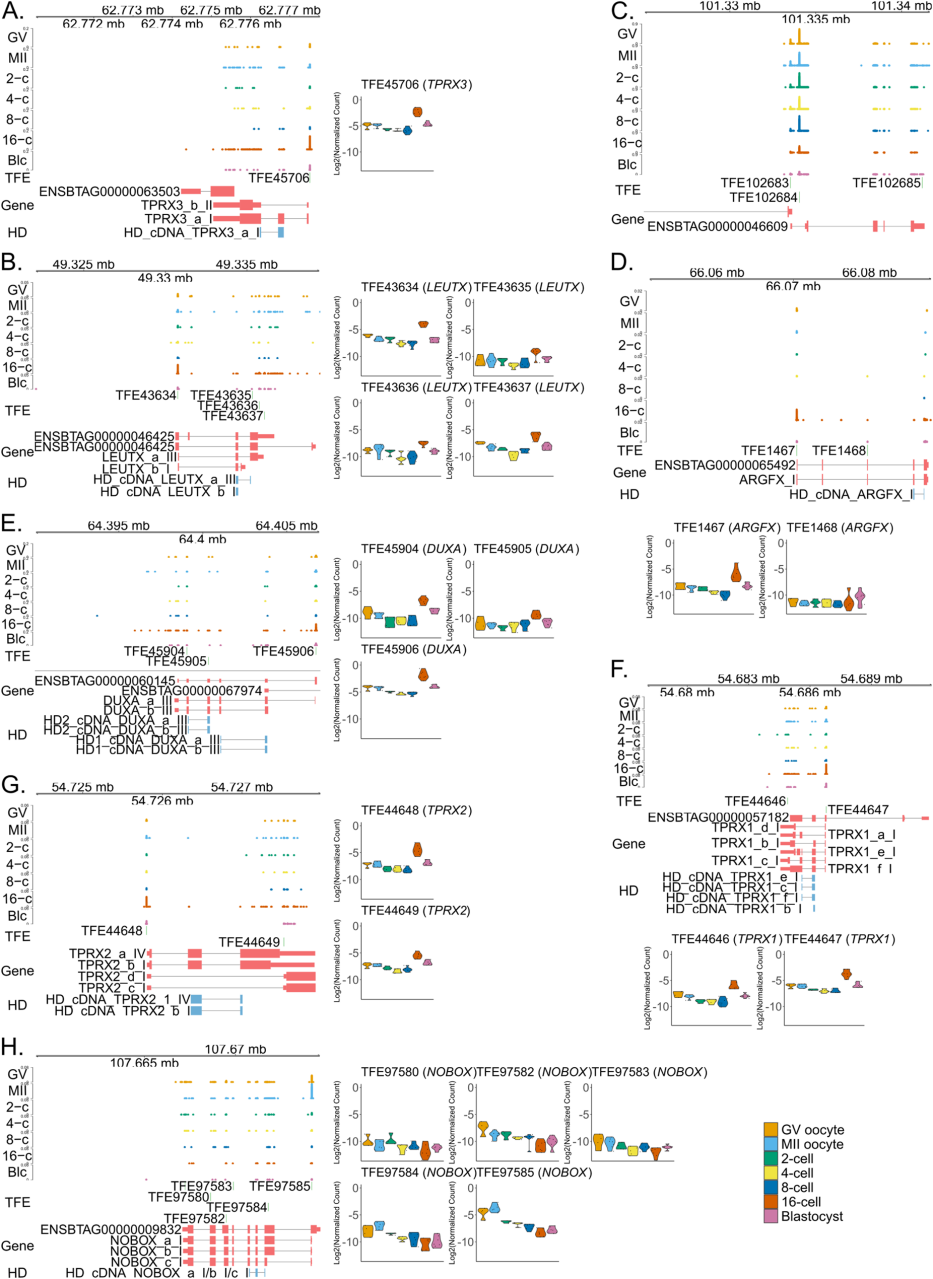
TFEs of selected genes with regard to their annotations. Read coverage (bigWig files), TFE peak (light green), gene annotation (pastel red), homeobox (light blue) tracks for (**A**) *TPRX3*, (**B**) *LEUTX*, (**D**) *ARGFX*, (**E**) *DUXA*, (**F**) *TPRX1*, (**G**) *TPRX2*, and (**H**) *NOBOX* and respective violin plots visualizing their dynamic expression. **C** Read coverage, TFE peak and gene annotation tracks for *DPPA3* (ENSBTAG00000046609). TFE and read coverage tracks are strand-specific and are found on the sense strand for *LEUTX* (TFE43634, TFE43635, TFE43636 and TFE43637), *DPPA3* (TFE102683 and TFE102683), *ARGFX* (TFE1467 and TFE1468) and *TPRX2* (TFE44648 and TFE44649), and anti-sense strand for *TPRX3* (TFE45706), *DUXA* (TFE45904, TFE45905 and TFE45906), *TPRX1* (TFE44646 and TFE44647), and *NOBOX* (TFE97580, TFE97582, TFE97583, TFE97584, TFE97585). TPRX3_a_I (OY970444), TPRX3_b_II (OY970448), LEUTX_a_III (OY970424), LEUTX_b_I (OY970425), ARGFX_I (OY970413), DUXA_a_III (OY970418), DUXA_b_III (OY970421), TPRX1_a_I (OY970431), TPRX1_b_I (OY970432), TPRX1_c_I (OY970433), TPRX1_d_I (OY970434), TPRX1_e_I (OY970435), TPRX1_f_I (OY970436), TPRX2_a_IV (OY970439), TPRX2_b_I (OY970441), TPRX2_c_I (OY970442), TPRX2_d_I (OY970443), NOBOX_a_I (OY970426), NOBOX_b_I (OY970427), and NOBOX_c_I (OY970429) represent alignments of cDNAs with the ENA accessions in parenthesis. Genomic coordinates from chromosomes 18 (**A**, **B**, **E**,** F**, **G**), 5 (**C**), 1 (**D**), and 4 (**H**) are shown. The Y-axis range of the read coverage track is 0–0.2 (**A, E**), 0–0.05 (**B**), 0–0.9 (**C**), 0–0.02 (**D**), 0–0.08 (**F, H**), 0–0.06 (**G**). HD_cDNA_ prefixes represent the alignments of ORFs encoding homeodomains for the respective genes. GV: GV oocyte; MII: MII oocyte; 2-c: 2-cell; 4-c: 4-cell; 8-c: 8-cell; 16-c: 16-cell; Blc: Blastocsyt; HD: Homeodomain

*TPRX3* is a bovine and porcine-specific gene [29], and we observed one TFE related to *TPRX3*, TFE45706, which was lowly expressed until the EGA and peaked in expression during the EGA. TFE45706 uncovered a potential TSS upstream of our cDNA-based *TPRX3* structure [16]. Detected TFE45706 should give rise to the transcript that codes for homeodomain (Fig. 4A). Similarly, the expression of *LEUTX* was also observed across all stages, with the 16-cell stage marking its highest expression level (Fig. 4B) in contrast to its reported expression profile marking the peak at the 8-cell stage [29], shown using a dataset from a previous work [5].

TFE-based analysis provides a transcriptomic overview that considers the impact of unannotated or potentially misannotated features. One example is *DPPA3*, as our TFE analysis indicated that the TFE102684 was in an intronic region with respect to the gene model ENSBTAG00000046609 (Ensembl Release 112) (Fig. 4C). This TFE highlights a previously unexplored exon that requires experimental validation and emphasizes the impact of our approach on RNA-seq quantification.

We further explored the expression of other PRDL homeobox genes. Our analysis revealed two TFEs for *ARGFX* (TFE1467 and TFE1468); one of the TFEs positioned at the 5’ end reached its peak level at the 16-cell stage, while the other showed very low expression (Fig. 4D). Since TFEs are derived from the 5’ ends of mRNAs, the detected TFE1467 suggested that the full-length *ARGFX* transcripts contained the homeodomain encoding region. Unlike *ARGFX*, *DUXA* had more than one TFE showing variable expression with highest levels at the same stage (Fig. 4E). In addition to TFE45906, which likely represents a transcript encoding double homeodomain, TFE45905 also demonstrated a slight increase following EGA (lower track in light blue; Fig. 4E). Interestingly, the putative full-length *DUXA* transcript was expressed across all stages with a sharp increase observed at the 16-cell stage, while another *DUXA* transcript (TFE45905) was exclusively expressed at the 16-cell stage.

The TFEs detected for *TPRX1* were located around our molecular cloning-based annotations rather than in the first two exons of ENSBTAG00000057182, which might represent a transcript that is active in a specific tissue or cell type but not during early embryo development (Fig. 4F). The TFEs corresponding to *TPRX2* were expressed at low levels and markedly increased their expression following EGA (Fig. 4G). *NOBOX* is another PRDL homeobox gene primarily expressed in oocytes (Fig. 4H). Interestingly, in addition to *NOBOX*, we also observed the expression of *DUXA*, *LEUTX*, and *TPRX*s, as well as, to a lesser extent, *ARGFX* in oocytes (Fig. 4).

We extended our gene selection by including previously described PRDL genes [30]. We categorized genes as maternal or zygotic based on whether their expression was predominantly observed in the heatmap in oocytes or in embryonic stages, and we showed that *ALX1*, *ALX3*, *ARX*, *HESX1*, *HOPX*, *MIXL1*, *NOBOX*, *PHOX2A*, *PITX3*, *SHOX*, *SHOX2*, *UNCX*, *VSX1* and *VSX2* function as maternal, whereas *ARGFX*, *DUXA*, *LEUTX*, *OTX2*, *PHOX2B* and *TPRXs* function as zygotic PRDL genes (Supplementary Fig. 3).

Our results underscore the importance of PRDL homeobox TF genes in bovine embryo development, given their involvement as maternal and embryonic factors, the latter of which shows prominent activation after bovine EGA, suggesting their role in subsequent processes needed for developmental progression.

### Putative bovine EGA inducers and genes with mutual temporal regulation

**Fig. 5:**
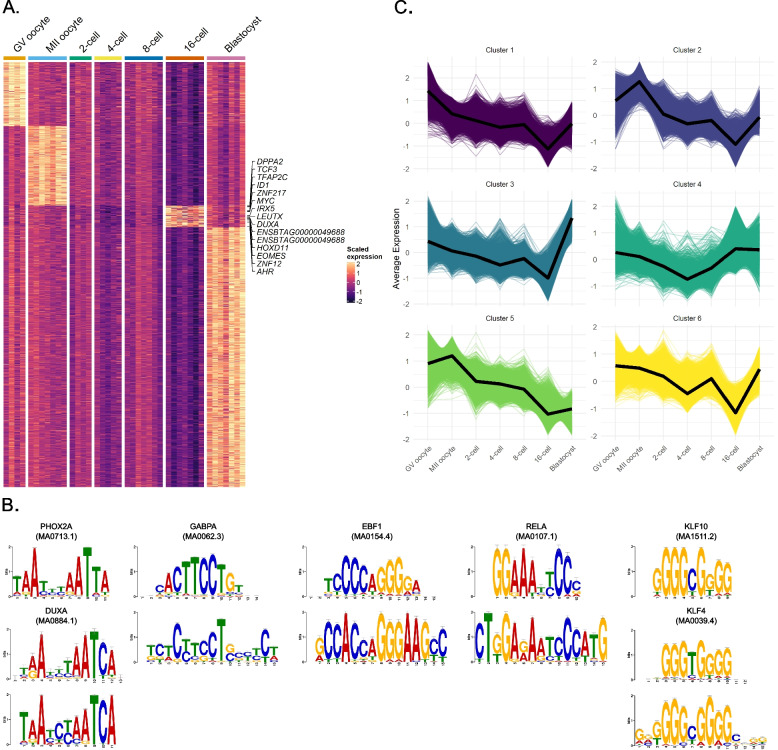
TFEs with distinct expression patterns and enriched de novo motifs around the promoters of 16-cell specific TFEs. **A** Heatmap of TFEs expressed exclusively at the GV oocyte or MII oocyte, or 16-cell or blastocyst stages. 16-cell-specific TFEs that correspond to bovine transcription factors [31] were labeled with their respective gene symbol or gene ID. Genes with more than one TFE were labeled once for each TFE they had. **B** De novo motifs discovered around the marker TFEs for the 16-cell stage are shown at the bottom, with the target motifs of their respective binders shown above them. The de novo motifs were shown in a decreasing order from left to right based on their ranks assigned by XSTREME. **C** K-means clustering of TFEs based on their dynamic expressions

Some TFEs were exclusively expressed at specific stages in early bovine development, suggesting that they may have stage-specific roles. By intersecting identified differentially expressed (DE) TFEs with those exclusively expressed at one stage, we detected marker TFEs for each developmental stage (Fig. 5A). Exceptions are the 2-, 4-, and 8-cell stages whose expression profiles showed close similarity and minimal DE TFEs (Figs. 1C and Fig. 3C, D). At the GV oocyte, MII oocyte, 16-cell stage embryo and blastocyst stages, we identified 588, 730, 197 and 2,379 stage-specific TFEs that correspond to 339, 155, 75 and 1400 known genes, respectively. The number of unannotated marker TFEs at each stage were 221, 572, 117 and 609. Furthermore, to characterize developmental stages with TFs, we examined the TF genes present in the bovine genome [31] and demonstrated TFEs of TFs that are expressed in a stage-specific manner (Supplementary Fig. 4).

To identify EGA-specific genes, we focused on the 16-cell stage. Among the 16-cell stage-specific TFEs, one marked *DPPA2*, which, in contrast, is maternally provided in mice [32] (Fig. 5A; Supplementary Table 5). Other 16-cell marker TFEs included *IRX5*, *ZNF217*, and *LEUTX* (Fig. 5A), which were reported to exhibit open chromatin at their regulatory regions near the promoter and distal sites in human 4-cell embryos prior to EGA [33]. Another PRD-like class gene, *DUXA*, was also identified as a marker TFE for this stage, specifically TFE45905 that was found downstream of TSS of full-length *DUXA* (Fig. 4E). ENSBTAG00000049688, which is a *ZSCAN4* paralogue, had two TFEs exclusive to the 16-cell stage.

To reveal factors that might induce EGA by binding around the promoters of genes upregulated during the 8-to-16-cell transition, we extracted the promoters of TFEs unique to the 16-cell stage and generated de novo motifs using XSTREME [34] (Fig. 5B; Supplementary Table 6, 7). The top de novo motif was similar to known binding motifs of DUXA, a zygotic PRDL TF, and PHOX2A, a maternal PRDL TF. The latter TF showed a gradual decrease in its mRNA expression levels from oocyte stages to the blastocyst stage (Supplementary Fig. 3 and 5A). Similarly, ALX1 and UNCX, other maternal PRDL TFs that were detected as potential binders of the same de novo motif, showed higher expression in oocytes with the former dropping its levels rapidly between GV and MII oocyte stage (Supplementary Table 7; Supplementary Fig. 5A). The next highest ranked motif matched the GABPA consensus site. *GABPA* was expressed in oocytes and reached its maximum levels at the 16-cell stage (Supplementary Fig. 5B). The EBF1 binding motif was also found to be enriched around the TSS of 16-cell-specific TFEs. This gene was expressed at higher levels before EGA (Supplementary Fig. 5C). Another identified de novo motif exhibited a significant match (p-value: 1.76e-04) with RELA, a subunit of NF-κB, whose mRNA levels were mostly higher in oocytes and pre-EGA embryos (Supplementary Fig. 5D). We also identified enriched binding motifs for *KLF4* and *KLF10*, which increased expression at the 16-cell stage and were also expressed in earlier stages, including oocytes (Supplementary Fig. 5E; Supplementary Table 3). Even though we detected OTX2 as another PRDL homeobox protein [18] that could bind the de novo generated motif (Supplementary Fig. 5F), *OTX2* exhibited very low expression levels before the blastocyst stage (Supplementary Fig. 5G). Notably, K50-type PRDL TFs other than OTX2, such as LEUTX and TPRX1, have similar binding motifs [18], making bovine LEUTX and TPRXs candidate EGA-inducing TFs. Our promoter analysis proposes putative factors that drive bovine EGA.

Next, to explore genes with possible shared regulation based on their temporal expression patterns, we performed k-means clustering of TFEs (Fig. 5C). Cluster 1, as the cluster with the highest expression in GV oocyte among all clusters, contained *RFX5, RFX7*, *FIGLA*, *TCF3* and *TCF12*, whose binding motifs were shown to be enriched in open chromatin unique to GV oocyte [26]. Cluster 4, with average expression peaking during EGA, included the PRDL homeobox genes *ARGFX*, *DUXA*, *LEUTX*, *TPRX1*, *TPRX2*, and *TPRX3*, which we recently confirmed to be expressed in bovine embryos through molecular cloning [16]. An increase in expression during the 4-to-8-cell transition, with a subsequent sharp drop following EGA, was observed in cluster 6, which included *KLF7*, *KLF15*, *SP1*, *SP4*, *SP8* and *HOXC13*. The binding motifs recognized by the products of these genes were previously shown to be most enriched in open chromatin at the 8-cell stage in bovine [26]. The clustering analysis suggests mutual temporal regulation of genes with particular roles.

## Discussion

In this study, we identified maternal (*ALX1*, *ALX3*, *ARX*, *HESX1*, *HOPX*, *MIXL1*, *NOBOX*, *PHOX2A*, *PITX3*, *SHOX*, *SHOX2*, *UNCX*, *VSX1* and *VSX2*) and zygotic (*ARGFX*, *DUXA*, *LEUTX*, *OTX2*, *PHOX2B* and *TPRXs*) PRDL TFs with promoter-based quantification. The expression of maternal and zygotic genes was observed primarily in oocytes and embryonic stages following fertilization, respectively. We identified transcripts only present at the 16-cell stage, providing a transcriptomic fingerprint that defines this stage while highlighting their roles in guiding subsequent stages following EGA. Furthermore, investigation of promoter regions of these genes marked PRDL class TFs, DUXA, PHOX2A, ALX1, UNCX, LEUTX, and TPRXs as well as other class TFs, GABPA, EBF1, RELA, and KLFs as putative bovine EGA-inducing factors whose mRNA expression was confirmed in early development.

During early embryo development, RNA amounts fluctuate between developmental stages due to the degradation of maternal transcripts as part of MZT and the initiation of EGA. In our study, we observed a continuous decrease of poly(A)-tailed RNA until the 16-cell stage. Our finding is in line with an earlier study, which reported a continuous decrease of poly(A)-tailed mRNA content until the 8-cell embryo stage in bovine [35], indicative of maternal mRNA degradation (Fig. 2A). The higher extent of decrease between 8- and 16-cell stage we showed implies successful poly(A)-tailed RNA degradation with possible involvement of EGA, an event shown to be required for the degradation of hundreds of transcripts in bovine 8-to-16-cell stage embryos [6]. While we observed a reversal of this trend of decrease in poly(A)-tailed RNA content at the blastocyst stage, the previous study, due to differences in embryonic stage sampling, observed this reversal at the morula stage [35].

Differential expression analysis with the spike-in based normalization demonstrated the transcriptional dynamics at the TFE-level, revealing minimal upregulation of TFEs at the 2-cell stage (Fig. 3B), which was reported to be transcriptionally active through ^35^S-UTP incorporation experiment, however, with the highest variability between the cells [36]. This stage was accompanied by the downregulation of the highest number of TFEs, a clear indication of maternal poly(A)-tailed RNA degradation following fertilization as a major event (Fig. 3B). However, there were also upregulated TFEs, often with high fold change values but statistically less significant which is due to high variation between embryos. The high interindividual variability observed may reflect differences in the developmental potential of these embryos, especially since most 2-cell stage embryos fail to reach the blastocyst stage [37]. A recent study suggests that cell fate may already be determined at the 2-cell stage [38]. Variations in TFEs expression at this stage could serve as potential biomarkers of developmental competence; however, our data alone are insufficient to support this, and further functional studies are needed. The number of DE TFEs observed at the 8-to-16-cell transition validated earlier reports regarding the timing of bovine IVF EGA [5, 12], and confirmed that bovine IVF EGA timing is distinct from that of human EGA [9] (Fig. 3E). Progression from the 8- to the 16-cell stage marked the most pronounced downregulation of genes among all consecutive comparisons, which is suggestive of EGA effect on RNA degradation as evidenced by transcriptionally-inhibited embryos in another study [6].

The genomic positions of TFEs in relation to the gene model may indicate different events. In TFE computational analysis pipeline [23], mapped RNA-seq reads that are aligned with the first exons of assembled transcripts are retained [9]. The 5’ ends of such reads, termed TFEs, might reveal previously unannotated TSSs when compared with gene models, such as TSSs located in the 5’-UTR or upstream (Fig. 4A), within an intron (Fig. 4C), or in the 3’-UTR (Fig. 4C) of the coding gene model. However, if a TFE is retained due to its overlap with a false assembled transcript and is located in the 3’-UTR, it may reflect a degrading transcript that has undergone reverse transcription event. Therefore, TFEs found in the 3’-UTR could either be derived from a degrading transcript or an alternative isoform.

Promoter analysis enabled us to identify several potential bovine EGA factors in 8-to-16-cell stage transition. One such factor is a PRDL TF, PHOX2A, whose binding motifs were reported to be enriched in the promoters of genes upregulated in human parthenogenetic 4-cell embryos, suggesting the role of PHOX2A as a maternal factor [39]. In contrast to the reported low expression profile of *PHOX2A* throughout bovine early development [26], our study showed high levels of *PHOX2A* specifically in oocytes (Supplementary Fig. 3 and 5A). In the same study, PHOX2A binding motif was found to be enriched exclusively in the open chromatin of bovine 8-cell embryos [26], suggesting a role before EGA commences. Given the abovementioned findings and the close match of its binding motif with the constructed de novo motif TAAYCYAATCA, we identify PHOX2A as a maternal factor with a putative role in promoting bovine EGA. UNCX and ALX1 are other maternal PRDL TF that exhibit a similar temporal expression pattern and binding motif with PHOX2A, implying their similar roles. Carryover of the proteins present in fully grown oocytes to later stages, including the blastocyst stage, has been recently observed in mice [40]. If a similar phenomenon persists in bovine, specifically for maternal PRDL TFs, it implies a potential mechanism for these TF in driving EGA. DUXA, a zygotic PRDL TF as a potential binder of the same motif, had its binding motif identified in the open chromatin of not only pre- but also post-EGA embryos [26], suggesting its role during and after EGA, while further investigation is needed. Another factor is GABPA whose binding motif was identified to be enriched in the promoters of genes definitive for human 8-cell-like cells as determined by a combination of pseudotime trajectory and motif analysis [41], pointing to its potential involvement in both human and bovine EGA. Given its steady expression in each stage alongside with an increase following bovine EGA (Supplementary Fig. 5B), GABPA is likely to have multiple roles similar to mouse, where GABPA does not only regulate EGA but also contributes to epiblast formation [42]. EBF1 was identified as a pioneer factor in mouse B cell lineage [43], a transcript factor capable of binding to DNA sequences wrapped and accessible on nucleosomes [43]. This suggests a role for EBF1 in initiating chromatin opening to allow binding by other factors, which needs further confirmation also in preimplantation development. If its pioneer factor function is conserved in bovines, as a maternal factor, EBF1 can potentially be one of the initial factors to drive early embryo development before or during EGA. RELA, a subunit of NF-κB, whose inhibition in pre-EGA mouse embryos prevented them from reaching the blastocyst stage [44], had its mRNA levels higher in early zygotic stages and its binding motif found in 16-cell specific gene promoters, implying that it could have an effect on EGA in bovines. Similar to our findings in bovine IVF embryos, in which *KLF4* and *KLF10* were upregulated at the 16-cell stage (Supplementary Table 3), they were also shown to increase their expression as well as chromatin accessibility following EGA in humans, namely at the 8-cell stage [33]. Specific residues of TFs are primarily involved in DNA binding, such as the residue 50 in the homeodomain of PRDL TFs [8]. Depending on this residue, PRDL TFs have been categorized into K50-, Q50- or R50-type TFs [18, 45]. Despite the binding motif enrichment of K50-type PRDL TF OTX2 we discovered around the promoters of the 16-cell stage-specific genes (Supplementary Fig. 5F), its expression profile suggested that its impact on bovine early embryo development might not be identical to that of humans, in which high *OTX2* mRNA abundance was observed before blastocyst formation [46]. Yet, the identified de novo motif CTTTAATCCAATTTT contains the subset sequence of TAATCC, which was experimentally confirmed to be the target sequence of another K50-type PRDL TFs, LEUTX and TPRX1, resulting in transcription activation [18]. This raises the possibility that other bovine K50-type PRDL TFs, LEUTX and TPRXs, may bind to this de novo motif during EGA. Taken together, maternal Q50-type (PHOX2A, UNCX, and ALX1), zygotic Q50-type (DUXA), and zygotic K50-type (LEUTX and TPRXs) PRDL TFs alongside with proteins from other classes such as GABPA, EBF1, RELA, and KLF factors exhibited mRNA expression in pre-EGA stages with their binding motifs enriched in the promoters of transcripts activated immediately after EGA. Therefore, we identify these factors as putative EGA-promoting TFs in bovine.

The strength of our study lies in leveraging 5’ transcript ends for more accurate quantification; however, this strategy requires losing 3’ end information from transcripts, which may contain elements involved in post-transcriptional regulation during early embryonic development. For this reason, our data do not allow exploration of 3’-UTR-mediated regulation or identification of transcript isoforms with varying 3’-UTRs. Since we sampled single oocytes and embryos, our study does not capture cellular heterogeneity among blastomeres or the single-cells from the blastocyst stage. In this study, we selected potential EGA-inducing factors based on the detection of transcripts encoding these factors. However, our transcriptomics-focused approach may miss the possible involvement of proteins that originate from oocytes persisting until EGA but are lacking in mRNA form. While we do not directly discover EGA-inducing TFs, our study provides a more robust view of the transcriptional dynamics of key TFs and highlights the strength of 5’-end profiling in exploring putative TF that may promote EGA. The functional validation experiments will be necessary to confirm the roles of these factors in bovine EGA.

## Conclusions

In summary, our study provides candidate regulatory elements and TFs for bovine EGA. We performed 5’-end targeted single oocyte/embryo sequencing, which allowed us to distinguish between the degrading maternal transcripts and novel embryonic transcripts. We identified two major stages of transcript degradation first happening at the 2-cell stage, and second occurring at the 16-cell stage when the EGA was initiated. Moreover, our TFE-based quantification demonstrated the transient surge in the expression of PRDL homeobox genes at the time of EGA. Our analysis allowed us to find developmental stage-specific markers. De novo motif analysis of the 16-cell stage-specific markers revealed Q- and K50-type PRDL TFs (DUXA, PHOX2A, UNCX, ALX1, LEUTX and TPRXs) as well as GABPA, EBF1, RELA and KLF factors as potential key regulators of the bovine EGA.

## Methods

### Oocyte collection, in vitro maturation and fertilization, embryo culture and STRT-N RNA-seq library preparation

Holstein Friesian breed bovine oocytes were collected, matured in vitro, fertilized in vitro and cultured under hypoxia conditions as described earlier [28], in collaboration with the Estonian University of Life Sciences (Tartu, Estonia). STRT-N RNA-seq libraries were prepared as outlined previously [28] and sequenced at the Biomedicum Functional Genomics Unit (FuGU), University of Helsinki (Helsinki, Finland).

### Preprocessing of RNA-seq libraries

Base call (BCL) files from RNA-seq run, ARS-UCD1.3 *Bos taurus* genome assembly and ARS-UCD1.3.112 annotation file from Ensembl were used as inputs to run STRTN_bTau_ens112.sh from https://github.com/baryasar/bTau_Embryo_TFE with options -a ens and -s 8M3S75T6B at the High Performance Computing Center at the University of Tartu [47] as outlined [23]. The STRTN_bTau_ens112.sh script, an optimized version of STRTN.sh to work with the ARS-UCD1.3 genome and Ensembl v112 gene annotation, is designed to remove PCR duplicates using Picard MarkDuplicates [48] with the BARCODE_TAG = RX option, collapsing reads with the same mapping positions and UMIs [23]. The hisat2_extract_splice_sites.py and hisat2_extract_exons.py scripts were piped to grep -v ^NKLS for extracting splice sites and exons, respectively. Similarly, sequences with NKLS prefixes were filtered from the ARS-UCD1.3 genome FASTA file. To run the TFE analysis, the script STRTN-TFE.sh from https://github.com/gyazgeldi/STRTN was executed.

### Integrating cDNA sequences of ARGFX, DUXA, LEUTX, NOBOX, TPRX1, TPRX2, and TPRX3 in the bovine annotation file

The genome index of the ARS-UCD1.3 FASTA file was created using GMAP [49] version 2018–07-04 with the gmap_build tool. The cDNA sequences of the seven PRDL TF genes [16] were used to create GFF3 files using gmap with the option -f 2. The resulting GFF files for each gene were merged and converted to GTF using gffread [50] with the option -T. The merged GTF file containing PRDL homeobox genes were then further merged with the ARS-UCD1.3.112 annotation file. For generating an annotation file compatible with the pipeline, gtfToGenePred utility [51] was used with the options -genePredExt -ignoreGroupsWithoutExons and a placeholder was added in the first field using awk. This final file was placed in the src/directory and used for extracting the transcript ID and gene name associations, a file that was also placed in the src/directory by the STRTN_bTau_ens112.sh script.

### Statistics and reproducibility

TFE counts were imported to R (v.4.4.1) and processed using DESeq2 (v.1.44.0) with RNA spike-in controls being used for normalization. Outlier, low-quality and negative control samples, namely samples 1, 11, 13, 23, 26, 37, 41, and TFEs with zero counts were removed. Fluctuating TFEs were identified by calculating coefficients of variation followed by fitting a gamma generalized linear model (GLM) to the spike-in controls using the R package MASS (v.7.3.61) [52]. The technical variation of the expression was modeled based on the variation of spike-in RNAs, and then the significance of technical plus biological variation of the non-spike-in expression was tested [53]. The top 20,000 TFEs with the highest CV^2^ (coefficient of variation squared) and significant adjusted p-values (< 0.05) were selected. Variance stabilizing transformation (VST) values of the selected TFEs were used to compute pairwise Pearson correlations and visualized as a heatmap with pheatmap (v.1.0.12).

The file containing TFE counts with their annotations was used to calculate the proportions of genomic annotations by stage. First, TFE counts were summed after grouping by annotation. Then, the percentages of TFE counts for each annotation group and sample were calculated by dividing the summed counts by the total number TFE counts in each sample. The obtained percentages were averaged by group. Nine annotations were reduced to six by summing their percentages and merging the following definitions: ‘Coding 5’-UTR’ and ‘Coding upstream’ into ‘Upstream and 5′-UTR of coding’; ‘Coding 3’-UTR’ and ‘Coding CDS’ into ‘CDS and 3′-UTR of coding’; ‘Noncoding 1st-exon’ and ‘Noncoding upstream’ into ‘Upstream and 1 st exon of noncoding’.

In order to calculate relative poly(A)-tailed RNA content, the total number of mapped reads per sample were normalized with the total number of mapped 5’-end spike-in reads, which were averaged by developmental stage. The average relative values were then divided by that of GV oocyte stage that was set to one.

For differential expression analysis, DESeq2 (v.1.44.0) package in R was used. The TFE counts and samples that were filtered as abovementioned were used for running the DESeq() function with the default Wald test to estimate differential TFE expression across developmental stages. The design formula incorporated each stage as a factor. The results were extracted using the results() function, adjusting the contrast between two consecutive stages, with the later in the developmental timeline being compared to the earlier stage. Filtering based on adjusted p-value < 0.05 was applied, with log2 fold change greater and less than zero used to select up- and downregulated TFEs, respectively.

Gene Ontology (GO) enrichment analysis was performed using the clusterProfiler (v.4.12.6) [54] function enrichGO(), where ENSEMBL, org.Bt.eg.db, and Biological Process (BP) were specified for key type, organism database and subontology group, respectively. Significantly up- and downregulated (adjusted *p*-value < 0.05, log2 fold change > 0 or < 0) TFEs for each consecutive stage comparison were used for the analysis, with the default option being selected for the universe argument to ensure a consistent reference across comparisons, including all bovine coding genes available in org.Bt.eg.db.

Seurat (v.5.1.0) package was used for generating Uniform Manifold Approximation and Projection (UMAP) and heatmap plots. Normalization was performed using spike-in RNAs and UMAP was computed incorporating eight principal components derived from PCA.

To identify stage-specific marker TFEs, normalized expression data were grouped by stage and TFE. The median expression of samples was calculated for each TFE at each stage. The resulting data were used for filtering TFEs whose expression values were higher than −10 in the stage of interest but lower than −10 in all other stages. The TFEs were considered expressed if their log2-transformed normalized values were higher than −10. Log2-transformed normalized value of −10 was calculated to correspond approximately to nine copies of poly(A)-tailed RNA molecules (Supplementary Table 8). These filtered TFEs were intersected with those that showed significant upregulation (adjusted *p*-value < 0.05, log2 fold change > 3) as compared to the stage immediately preceding the stage of interest. An exception was made for the GV oocyte stage, in which case significant upregulation (adjusted *p*-value < 0.05, log2 fold change > 3) was assessed relative to the MII oocyte stage. Scaled data were used to create a heatmap of the marker genes with the DoHeatmap() function. ComplexHeatmap package (2.20.0) was used to mark TFs obtained from AnimalTFBD 4.0 [31].

K-means clustering was computed using the kmeans() function on scaled data, with the following parameters: centers = 6, nstart = 1000 and iter.max = 20.

For visualizations, R packages ggpubfigs [55] (v.0.0.1) and viridis [56] (v.0.6.5) providing colorblind-friendly color palettes were used. For genomic data visualization, R package Gviz [57] (v.1.48.0) was used.

### De novo motif construction using promoters around TFEs

TFEs specific to the 16-cell stage were selected if TFEs were significantly upregulated (adjusted *p*-value < 0.05, log2 fold change > 0) relative to the 8-cell stage and had log2-transformed normalized values higher than −10 for the 16-cell stage and lower for other stages (Supplementary Table 8). Additionally, the TFEs that were identified as noisy by DeepTSS [58] were removed. In Python (v.3.7.9) main.py script of DeepTSS was executed with the following inputs: TFEs BED files, ARS-UCD1.3 chromosome sizes and FASTA file and GERP conservation scores for *Bos taurus* ARS-UCD1.3 (Ensembl Release 112). The BED file of the selected TFEs was used to extract promoters located 400-bp upstream and 100-bp downstream of each TFE. FASTA files were obtained using bedtools [59] (v2.30.0) getfasta with the options -nameOnly -s -fi. To generate de novo motifs, XSTREME [34] tool, part of the MEME suite [60], was used with the default parameters on the web version. Out of the top 20 ranked de novo motifs, those with similar known motifs in the output of the XSTREME web version were selected (Supplementary Table 6). Only binding motifs of proteins present in the bovine gene annotation file were considered for further investigation. Among these, the binding motifs of proteins that ranked first with the two highest significance values were visualized as logos if they exhibited increased mRNA levels (Supplementary Fig. 6; Supplementary Table 7). Other de novo motifs were also visualized as logos if they had a significant match with a known motif from a protein encoded by a highly expressed mRNA (Supplementary Fig. 6; Supplementary Table 7).

## Supplementary Information

Additional file 1: Supplementary Figure 1. (A) Quality control metrics of sequenced STRT-N RNA-seq libraries. Supplementary Figure 2. (A-C) The top 10 significant biological process (BP) gene ontology terms for up- and downregulated genes in Fig. 3A, 3B, 3F, respectively. Developmental stage icons shown on the left represent the comparisons, with the later stage in the developmental timeline being compared to the earlier stage. The icons were retrieved from BioRender. Supplementary Figure 3. Heatmap demonstrating scaled expressions of TFEs of PRDL homeobox TF genes found in bovine. Supplementary Figure 4. Heatmap from Fig. 5A showing scaled expressions of GV oocyte-, MII-oocyte-, 16-cell-, or blastocyst-specific TFEs, with TFs marked. Bovine transcriptions factors [31] were labeled with their respective gene symbol or gene ID. Genes with more than one TFE were labeled once for each TFE they had. Supplementary Figure 5. Expression levels of TFEs corresponding to genes whose encoded products can bind to the de novo motif TAAYCYAATCA (A), TCTCYTCCTSCYYCT (B), GCCACCAGGGAAGCC (C), CTGGARAATCCCATG (D), and GVGGGGCGGGGCBGG (E) shown in Fig. 5B. (F) The de novo motif CTTTAATCCAATTTT identified around the marker TFEs for the 16-cell stage, with no potential binders of this motif showing high expression. (G) Expression levels of genes whose product can bind the de novo motif in (F).

Additional file 2: Supplementary Table 1. QC metrics for RNA-seq libraries. Supplementary Table 2. Stage-averaged percentages of aligned reads across TFE annotations. Supplementary Table 3. Differentially upregulated TFEs and their genomic positions and gene annotations for the 16-cell versus 8-cell stage. Supplementary Table 4. Differentially downregulated TFEs and their genomic positions and gene annotations for the 16-cell versus 8-cell stage. Supplementary Table 5. Sixteen-cell stage-specific TFEs and their gene names if corresponding to an annotated gene. If a TFE was identified in a genomic region without any annotation, it was marked as 'Unannot.' Supplementary Table 6. XSTREME results showing the de novo motifs ranked in the first 20 and contained known binders coded in bovine genome. Supplementary Table 7. Known binders of the de novo motifs presented in Supplementary Table 6. Supplementary Table 8. Converting the log2 normalized values back to their read counts and calculation of the estimated spike-in transcript counts.

## Data Availability

FASTQ files are available in EMBL-EBI BioStudies with the accession number S-BSST1958. The scripts required for STRTN pipeline and TFE analysis, and the R code used for creating figures can be accessed from [https://github.com/baryasar/bTau/_Embryo/_TFE] (https://github.com/baryasar/bTau_Embryo_TFE) and [https://github.com/gyazgeldi/STRTN] (https://github.com/gyazgeldi/STRTN).
