# Supplementary material for: RNA sequencing of mRNA 5’-ends reveals regulators of bovine embryonic genome activation
Source: BMC Genomics. 2025 Oct 14;26:910. doi: 10.1186/s12864-025-12110-x (PMC12522504; doi:10.1186/s12864-025-12110-x)
Supplement: Supplementary file 2 — Additional file 2: Supplementary Table 1. QC metrics for RNA-seq libraries. Supplementary Table 2. Stage-averaged percentages of aligned reads across TFE annotations. Supplementary Table 3. Differentially upregulated TFEs and their genomic positions and gene annotations for the 16-cell versus 8-cell stage. Supplementary Table 4. Differentially downregulated TFEs and their genomic positions and gene annotations for the 16-cell versus 8-cell stage. Supplementary Table 5. Sixteen-cell stage-specific TFEs and their gene names if corresponding to an annotated gene. If a TFE was identified in a genomic region without any annotation, it was marked as 'Unannot.' Supplementary Table 6. XSTREME results showing the de novo motifs ranked in the first 20 and contained known binders coded in bovine genome. Supplementary Table 7. Known binders of the de novo motifs presented in Supplementary Table 6. Supplementary Table 8. Converting the log2 normalized values back to their read counts and calculation of the estimated spike-in transcript counts. [file 12864_2025_12110_MOESM2_ESM.pdf]

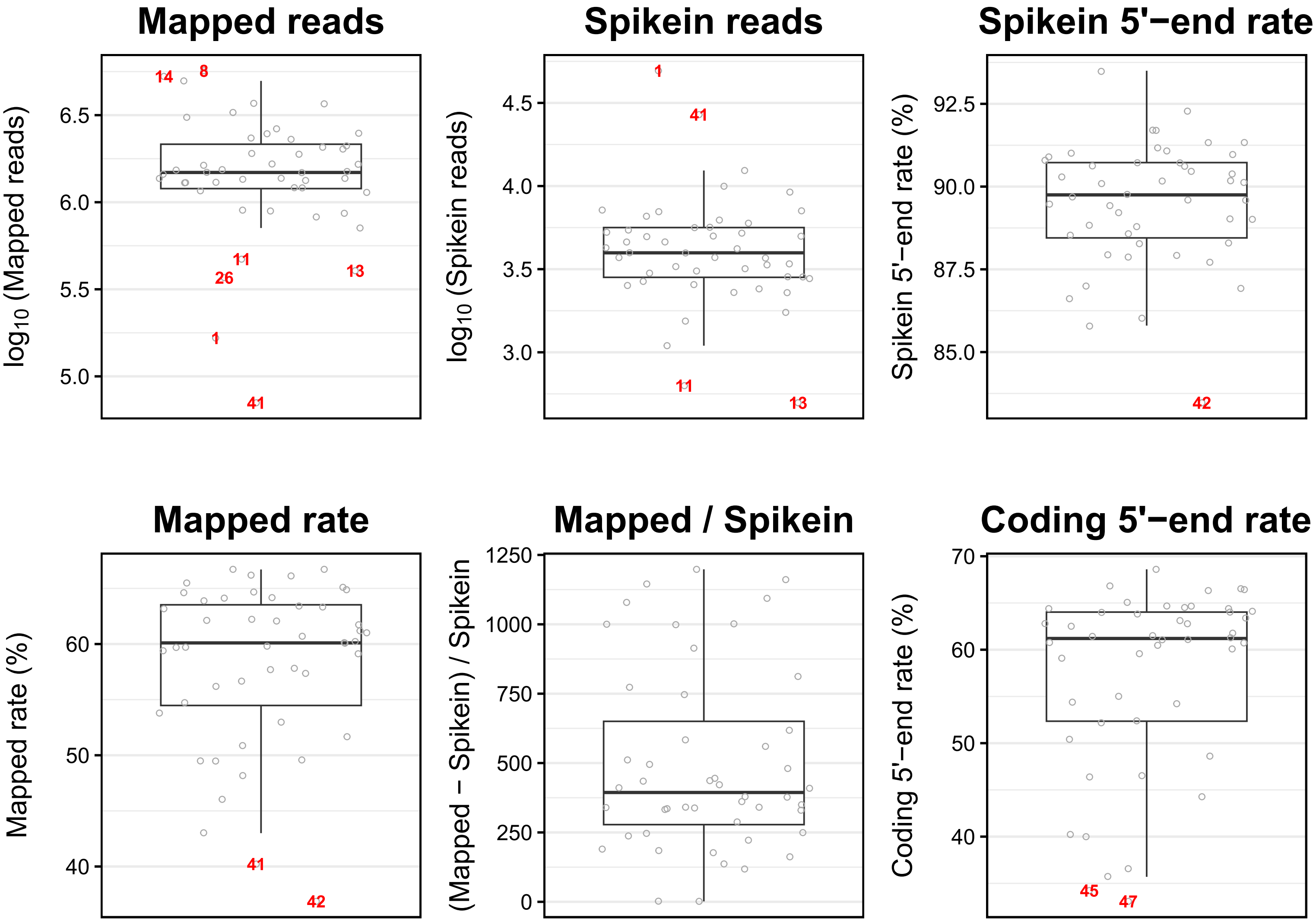

Supplementary Figure 1. Quality control metrics of sequenced STRT-N RNA-seq libraries.

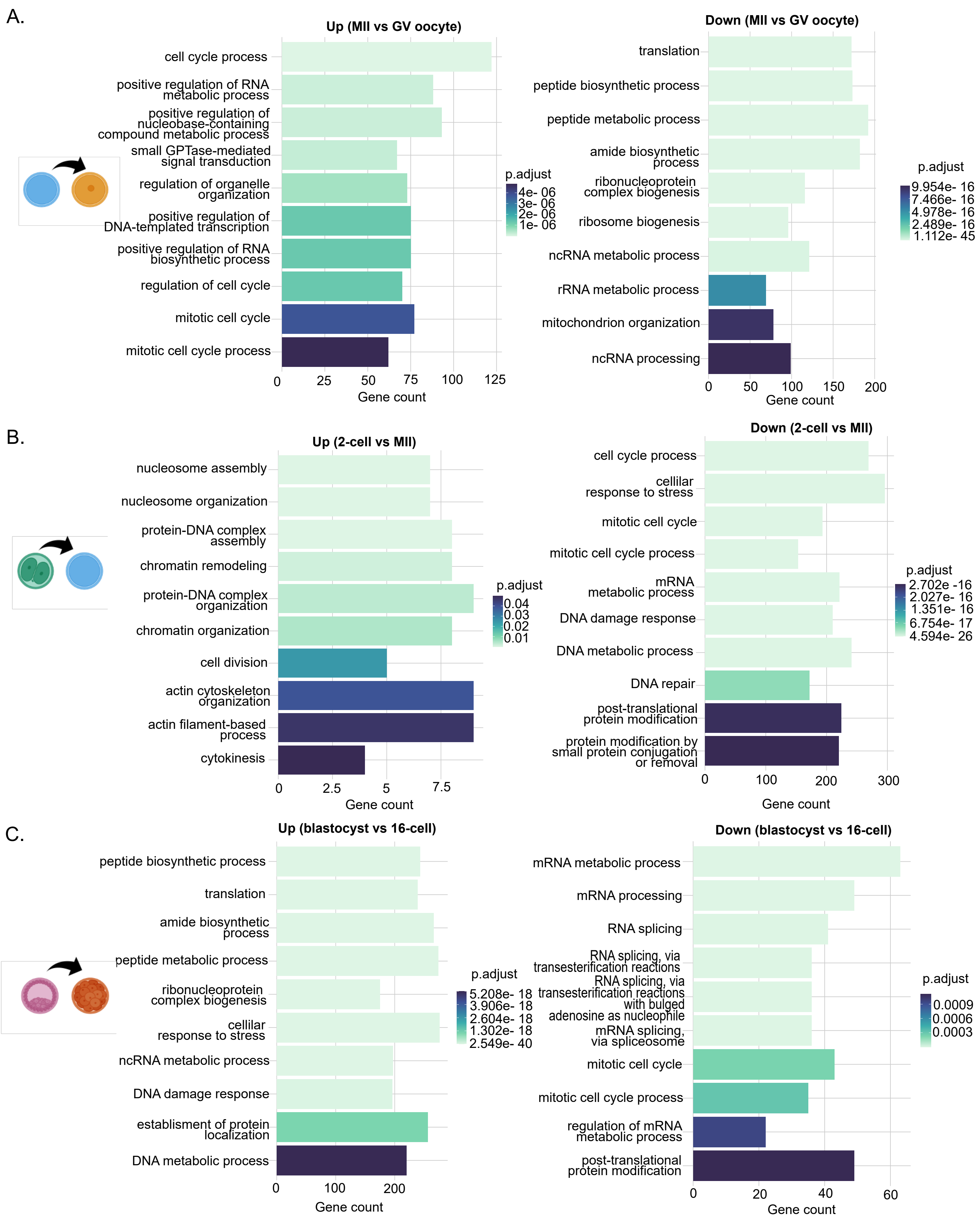

Supplementary Figure 2. (A-C) The top 10 significant biological process (BP) gene ontology terms for up- and downregulated genes in Fig. 3A, 3B, 3F, respectively. Developmental stage icons shown on the left represent the comparisons, with the later stage in the developmental timeline being compared to the earlier stage. The icons were retrieved from BioRender.

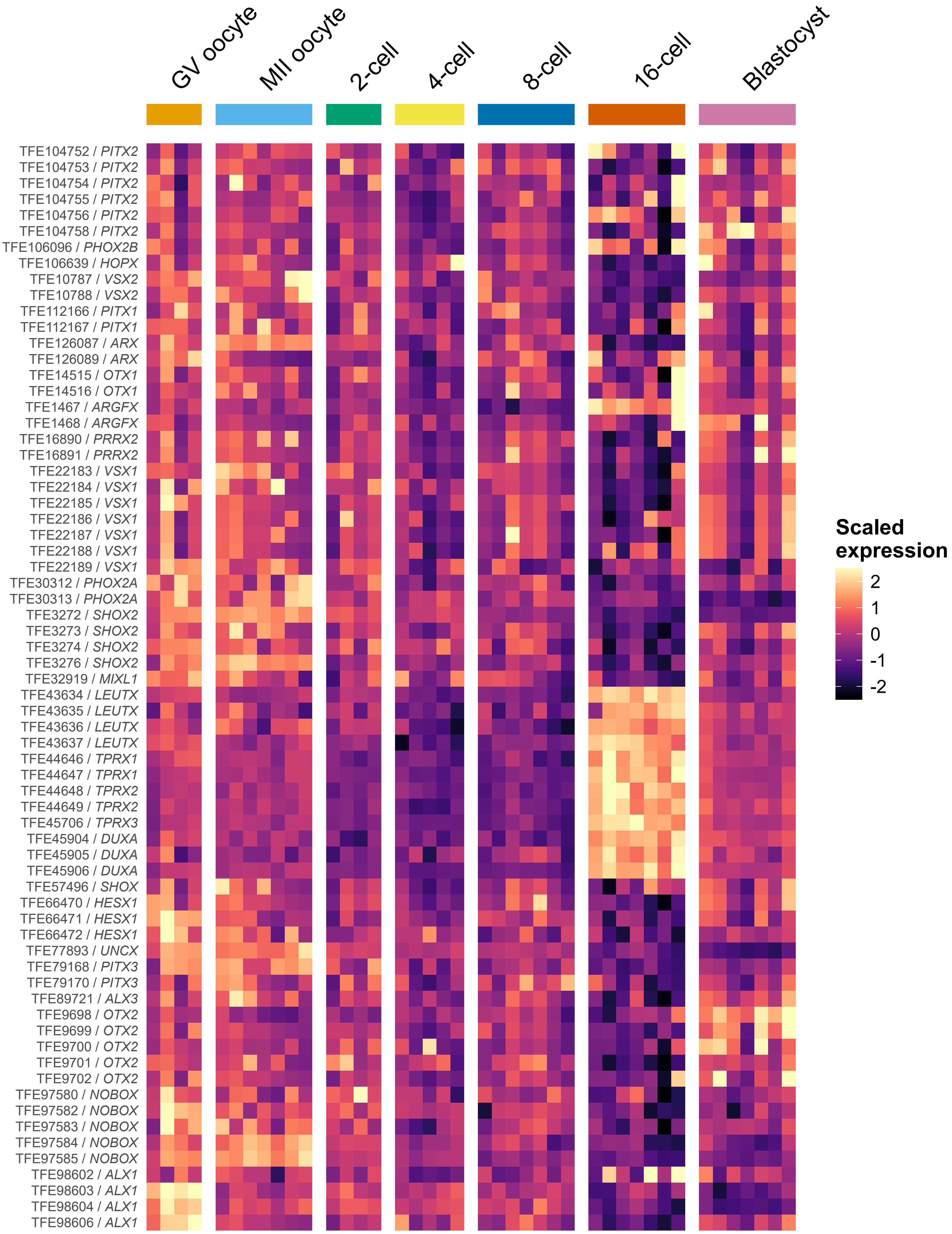

Supplementary Figure 3. Heatmap demonstrating scaled expressions of TFEs of PRDL homeobox TF genes found in bovine.

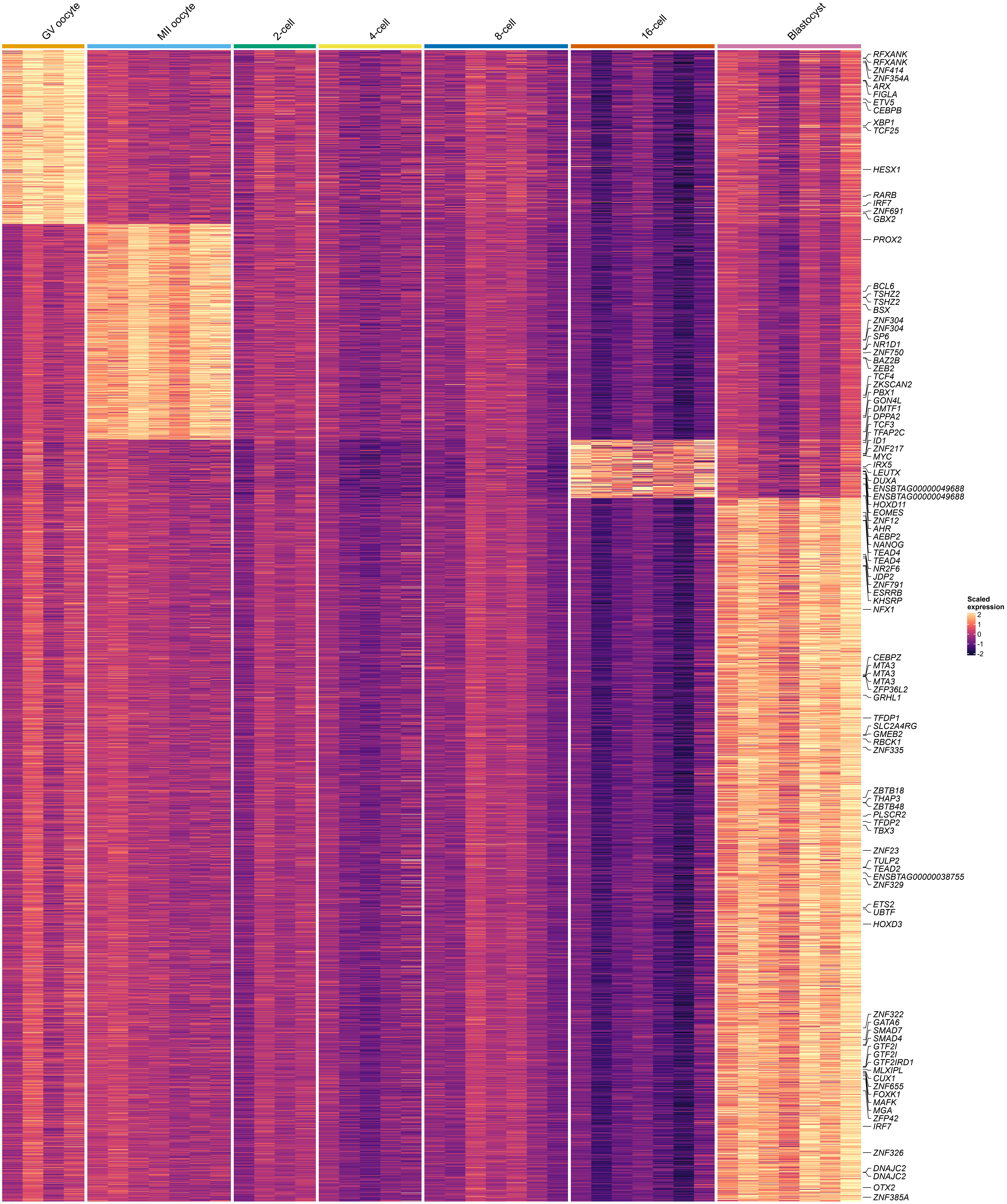

Supplementary Figure 4. Heatmap from Fig. 5A showing scaled expressions of GV oocyte-, MII-oocyte-, 16-cell-, or blastocyst-specific TFEs, with TFs marked. Bovine transcriptions factors[28] were labeled with their respective gene symbol or gene ID. Genes with more than one TFE were labeled once for each TFE they had.

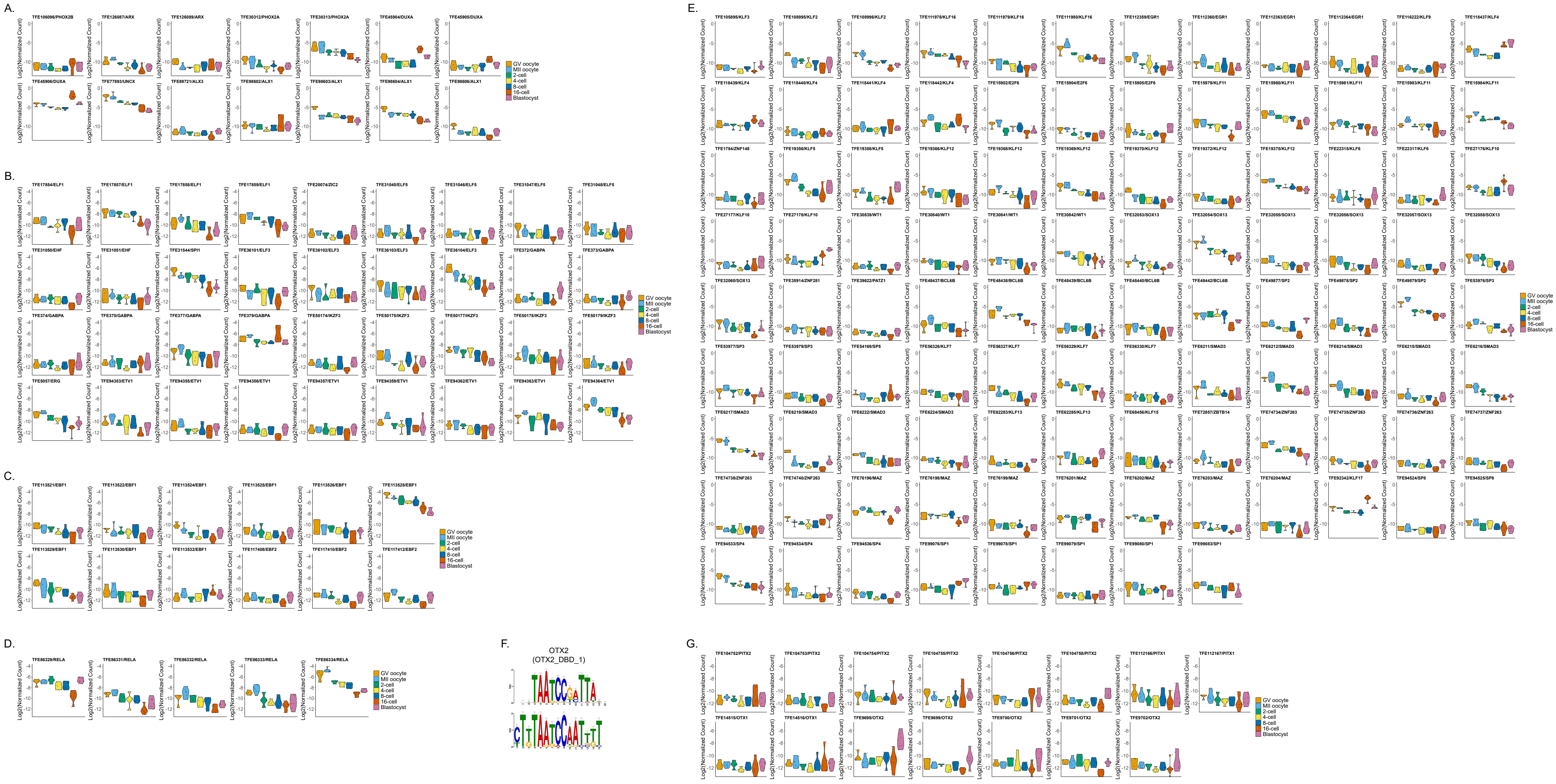

Supplementary Figure 5. Expression levels of TFEs corresponding to genes whose encoded products can bind to the de novo motif TAAYCYAATCA **(A)**, TCTCYTCCTSCYYCT **(B)**, GCCACCAGGGAAGCC **(C)**, CTGGARAATCCCATG **(D)**, and GVGGGGCGGGGCBGG **(E)** shown in Fig. 5B. **(F)** The de novo motif CTTTAATCCAATTTT identified around the marker TFEs for the 16-cell stage, with no potential binders of this motif showing high expression. **(G)** Expression levels of genes whose product can bind the *de novo* motif in **(F)**.
